# Rapid GC-MS method for screening seized drugs in forensic investigations: optimization and validation

**DOI:** 10.3389/fchem.2025.1559279

**Published:** 2025-06-10

**Authors:** Asma M. Askar, Auhood Y. Al Ali, Meera K. Khalifa, Ali A. Salem, Buthaina M. Alkhuwaildi, Iltaf Shah

**Affiliations:** ^1^ General Department of Forensic Science and Criminology, Dubai Police GHQ, Dubai, United Arab Emirates; ^2^ Department of Chemistry, College of Sciences, United Arabs Emirates University, Al Ain, United Arab Emirates

**Keywords:** seized drugs, GC-MS, screening, validation, optimization

## Abstract

**Indroduction:**

The escalating incidence of drug-related crimes requires rapid and reliable forensic methods for drug screening.

**Methods:**

This study develops and optimizes a rapid Gas Chromatography-Mass Spectrometry (GC-MS) method that significantly reduces the total analysis time from 30 to 10 minutes, facilitating faster judicial processes and law enforcement responses. Enhanced by optimizing temperature programming and operational parameters, the method efficiently shortens the run time while ensuring the accuracy essential for forensic applications.

**Results:**

Through systematic validation, the method demonstrated a limit of detection improvement by at least 50% for key substances such as Cocaine and Heroin, achieving detection thresholds as low as 1 μg/mL for Cocaine compared to 2.5 μg/mL with conventional method. Additionally, the method exhibited excellent repeatability and reproducibility with relative standard deviations (RSDs) less than 0.25% for stable compounds under operational conditions. Applied to 20 real case samples from Dubai Police Forensic Labs, the rapid GC-MS method accurately identified diverse drug classes, including synthetic opioids and stimulants, with match quality scores consistently exceeding 90% across tested concentrations.

**Conclusion:**

The method effectively reduces forensic backlogs, facilitating faster and more reliable drug screening essential for judicial processes.

## Introduction

The importance of advanced and dependable drug screening methodologies in forensic drug investigations has become increasingly critical as global incidences of drug trafficking and substance abuse escalated drastically in recent years.

Gas chromatography-mass spectrometry has been a key instrument in forensic drug analysis due to its high specificity and sensitivity (Black, Russell, Mayo and Aitcheson, 2023; Júnior et al., 2024; Liliedahl and Davidson, 2021). However, the conventional GC-MS techniques generally require extensive time for sample preparation and analysis. This duration often hinders rapid law enforcement responses and judicial processes, encouraging a need for faster analytical techniques (Capistran and Sisco, 2022). Recent advances have been developed to refine these methodologies to accelerate the screening process without sacrificing the analytical accuracy necessary as forensic evidence.

For instance, the development of a sensitive GC-MS protocol for synthetic opioids, optimizing instrumental parameters to analyze over 200 related compounds effectively (Sisco, Burns and Moorthy, 2021). Similarly, another study validated the GC-MS analysis of ecstasy tablets, emphasizing the quantification of psychoactive substances and detecting adulterants (Cunha et al., 2023). Rapid GC-MS techniques have also been implemented to accelerate the screening process for seized drugs, with some methods reducing run times to about 1 minute using special column through advanced temperature programming, proving effective for reducing case backlogs in forensic labs (Capistran and Sisco, 2024). Techniques such as Hollow Fiber Liquid-phase Microextraction (HF-LPME) combined with GC-MS have been used for sensitive and precise amphetamines detection in hair samples (Madia et al., 2022), while the use of GC/SIM-MS on a high-polarity GC capillary column has been employed for docosanol analysis in biological samples, validated against FDA guidelines with high recovery rates (Shankar et al., 2021). Comprehensive illicit substance profiling has also been integrated using GC-MS, ensuring evidence applicability through rigorous validation based on SWGDRUG guidelines (Radke, White, Loughlin and Cresswell, 2024). Furthermore, GC-MS/MS has shown high throughput and sensitivity for stimulants analysis in blood, presenting environmental and cost benefits over LC-MS/MS (Woźniak et al., 2020). Innovations such as the use of shorter and narrower columns in traditional GC-MS analysis effectively reduced the analysis time significantly for seized drug samples (Bloom, Sisco and Lurie, 2023). Another method enhanced the detection of cannabis-related compounds in biological samples with rigorous validation, emphasizing its high precision and accuracy (Paknahad et al., 2024). In medical applications, GC-MS has been effectively applied to quantify multiple cannabinoids in therapeutic cannabis oil, ensuring consistent and reliable results (Franzin et al., 2023). Lastly, the combination of solid-phase extraction with miniaturized mass spectrometers has facilitated the rapid on-site detection of various drugs in urine, highlighting the method’s high sensitivity and ease of use for field applications (Wu et al., 2024; Shah et al., 2019).

Despite these advancements, integrating rapid GC-MS technologies into forensic applications faces several challenges, including the need for comprehensive method validation and the adaptation of existing protocols to ensure reliability and reproducibility (Capistran and Sisco, 2024; Sisco, Burns and Moorthy, 2021). Systematic validation studies have begun to address these challenges by evaluating performance characteristics such as selectivity, sensitivity, precision, and accuracy in drug detection (Sisco, Burns and Moorthy, 2021). These studies provide a framework for the forensic community to adopt rapid methodologies with confidence. Despite these efforts, rapid GC-MS methods validated against SWGDRUG and UNODC standards using actual case samples remain limited.

This study introduces a developed and optimized rapid GC-MS method for drug screening that aims to significantly decrease analysis time from 30 min to 10 min. Utilizing the same 30-m DB-5 ms column. Upon its development, the methodology was subjected to a comprehensive validation protocol. Assessments were conducted on the repeatability and reproducibility of retention times, the accuracy of analysis identification, the determination of detection limits, and the evaluation of analysis carryover. Following the validation, the practical applicability of the method was examined through the analysis of adjudicated case samples sourced from the Dubai Police Forensic Laboratories, confirming its utility in authentic forensic contexts. This method maintains, and potentially enhances, the analytical accuracy and precision required in forensic drug investigations, thereby enhancing the efficiency of forensic analysis and supporting the broader objectives of law enforcement and public safety.

## Materials and methods

### Instrumentation

All the method development and the validation for rapid GC–MS work was conducted using an Agilent 7890B gas chromatograph (GC) system connected to an Agilent 5977A single quadrupole mass spectrometer (MSD) (Agilent Technologies, Santa Clara, CA. USA), equipped with a 7,693 autosampler, and an Agilent J&W DB-5 ms column (30 m × 0.25 mm × 0.25 μm). Helium (99.999% purity) was used as carrier gas at a fixed flow rate of 2 mL/min.

Data acquisition was completed using Agilent MassHunter software (MassHunter Workstation Software, GC–MS Data Acquisition, version 10.2.489, Agilent Technologies) and Agilent Enhanced ChemStation software (Version F .01.03.2357) for data collection and processing.

Retention times were extracted at the apex of a given peak in a chromatogram. Library searches were conducted with Wiley Spectral Library (2021 edition) and Cayman Spectral Library (September 2024 edition).

Rapid GC–MS analysis was conducted using the parameters described in Table 1. The conventional GC–MS method, developed in-house and employed by the Dubai Police forensic laboratories based on diverse literature over the years, was conducted using the same instrument to determine limits of detection (LOD) and for comparative purposes, with parameters detailed in Table 1. Previously, the conventional method has provided a reliable foundation for forensic analyses, ensuring consistent and comprehensive detection capabilities within the department’s strict operational requirements.

**TABLE 1:** Parameters for the optimized rapid GC-MS method and the conventional method.

Method parameter	Value (rapid method)	Value (conventional method)
Temperature Program	Initial: 120°C, ramp to 300°C at 70°C/min (hold 7.43 min)	Initial: 70°C, ramp (hold 3.0 min), ramp to 300°C at 15°C/min (hold 12 min)
Run time	10.00 min	30.33 min
Injection type	Split (20:1 fixed)	Split (20:1 fixed)
Inlet temperature	280°C	280°C
GC oven temperature	120°C	70°C
Ionization source	Electron Ionization, 70 eV	Electron Ionization, 70 eV
Transfer line temperature	280°C	280°C
Ion source temperature	230°C	230°C
Quadrupole temperature	150°C	150°C
Scan range	*m/z* 40 to *m/z* 550	m/z 40 to m/z 550
Sampling rate	N = 1	N = 1
Flow rate	2 mL/min	1 mL/min
Tune type	atune	atune

### Test solutions

#### General analysis mixture sets

A few test solutions, made either in-house or purchased from Cayman Chemical (Ann Arbor, MI. US) or Sigma Aldrich (Cerilliant, St. Louis, MO. USA), were used in this study. To develop a method manageable to a broad range of compounds of interest, two custom “general analysis” mixtures were prepared.

The first mixture contained Tramadol, Cocaine, Codeine, Diazepam, Δ9-Tetahydrocannabinol known as THC, Heroin, Alprazolam, Buprenorphine (Sigma Aldrich, Cerilliant, St. Louis, MO. USA), γ-Butyrolactone known as GBL and diphenoxylate (taken from cases) in methanol (99.9%, Sigma Aldrich, St. Louis, MO. USA) at an approximate concentration of 0.05 mg/mL per compound as listed in Table 2.

**TABLE 2:** Compounds, with respective molecular formulas, molecular masses, present in the mixture sets used for method development and the validation study.

Compound	Formula	Molecular Mass (Da)
Mixture set 1		
γ-Butyrolactone (GBL)^a^	C_4_H_6_O_2_	86.09
Tramadol	C_16_H_25_NO_2_	263.38
Cocaine	C_17_H_21_NO_4_	303.35
Codeine	C_18_H_21_NO_3_	299.37
Diazepam	C_16_H_13_ClN_2_O	284.74
Δ9-Tetahydrocannabinol (THC)	C_21_H_30_O_2_	314.46
Heroin	C_21_H_23_NO_5_	369.41
Alprazolam	C_17_H_13_ClN_4_	308.76
Buprenorphine	C_29_H_41_NO_4_	467.65
Diphenoxylate^a^	C_30_H_32_N_2_O_2_	452.59
Mixture Set 2		
Methamphetamine	C_10_H_15_N	149.23
3,4-Methyl​enedioxy​methamphetamine (MDMA)	C_11_H_15_NO_2_	193.25
Ketamine	C_13_H_16_ClNO	237.73
MDMB-INACA	C_20_H_28_N_4_O_3_	368.47
MDMB-BUTINACA	C_20_H_29_N_3_O_3_	359.47
Lysergic acid diethylamide (LSD)	C_20_H_25_N_3_O	323.44

^a^ Removed from validation study as they are seized drugs from cases.

The Second mixture contained MDMB-INACA, MDMB-BUTINACA (Cayman Chemical, Ann Arbor, MI, USA), Methamphetamine, 3,4-Methyl​enedioxy​methamphetamine known as MDMA, Ketamine and Lysergic acid diethylamide known as LSD (Sigma Aldrich, Cerilliant, St. Louis, MO. USA) in methanol (99.9%, Sigma Aldrich, St. Louis, MO. USA) at an approximate concentration of 0.05 mg/mL per compound as listed in Table 2.

### Case samples

To demonstrate the effectiveness of the rapid GC-MS method on real-world samples, 20 seized drug samples from different cases were analyzed. This included a variety of drugs, with 10 samples containing drugs in their solid form and another 10 trace samples collected from swabs of digital scales, syringes, and other drug-related items. Hence, to compare the identification capability of the rapid GC-MS method, all the samples were analyzed using both conventional and rapid GC-MS methods.

#### Extraction procedure

Liquid-liquid extraction procedures were applied to analyze both solid and trace samples for the presence of drugs. For solid samples, tablets and capsules were first ground into a fine powder using a mortar and pestle. Approximately 0.1 g of this powdered material was then added to a test tube containing about 1 mL of 99.9% methanol (Sigma Aldrich, St. Louis, MO, USA). The mixture was sonicated for approximately 5 min and then centrifuged to separate the phases. The clear supernatant liquid was carefully transferred into a 2 mL GC-MS capped vial to prepare it for analysis. In the case of trace samples, which involved collecting residues from drug-related items, swabs pre-moistened with 99.9% methanol (Sigma Aldrich, St. Louis, MO, USA) were used. The swabs were systematically rubbed across the surface of the items using a single-direction technique to maintain controlled pressure and prevent contamination. After swabbing, the tips of the swabs were immersed in approximately 1 mL of methanol and vortexed vigorously to ensure effective extraction of any analytes. This methanol extract was then transferred into a 2 mL GC-MS capped vial for subsequent analysis.

### Method development and validation

The general analysis mixture sets were utilized to develop and optimize the temperature program and flow rate of the rapid GC–MS method by trial-and-error process. Table 1 describes the optimized rapid GC–MS method of analysis for seized drugs.

Using the aforementioned drug mixture sets, the following method validation parameters were analyzed: retention time (RT) repeatability and reproducibility, limits of detection (LOD), analyte identification accuracy, and carryover. To evaluate RT repeatability and reproducibility, drug class mixtures mentioned in Table 2 were used and the intraday precision of the RT of all components of each mixture was determined by analyzing seven replicate analyses in a single day. In order to examine repeatability in the course of time, the interday accuracy and shift of the RTs were measured using seven replicates for each mixture on the first week, 1 week after, and 2 weeks later. The percentage relative standard deviation (RSD) between the replicate measurements was calculated and utilized to define the RT precision.

Then, the general analysis mixture sets were consecutively diluted in methanol to approach the method’s limits of detection (LODs) and compare it with the conventional GC–MS method. The lowest concentration that produced a chromatographic peak with a “Match Quality” score of at least 80% and a signal-to-noise ratio of at least 3:1 was determined as the approximate LOD concentration. The concentrations used for each mixture were 50 μg/mL, 30 μg/mL, 15 μg/mL, 5 μg/mL, 2.5 μg/mL, and 1 μg/mL. A comparison between the obtained LODs of the conventional GC-MS method and the Rapid Method is found in Table 3 *.*

**TABLE 3:** Retention Time Precent and Limit of Detection for Set mixtures one and two for rapid GC-M method and conventional GC-MS method.

Drugs	Retention Time	Percent RSD		Approximate limit of detection (LOD) (µg/mL)	
Standards	Average RT (min) (n = 21)	Intraday (n = 7)	Interday (n = 21)	Rapid GC-MS	Conventional GC-MS
Mixture set 1					
Tramadol	2.758 ± 0.005	0.197%	0.176%	2.5	5
Cocaine	3.189 ± 0.006	0.223%	0.177%	1	2.5
Codeine	3.631 ± 0.007	0.195%	0.203%	5	5
Diazepam	3.708 ± 0.007	0.217%	0.197%	2.5	2.5
Δ^9^-Tetahydrocannabinol (THC)	3.733 ± 0.008	0.224%	0.209%	2.5	5
Heroin	4.193 ± 0.009	0.211%	0.210%	5	15
Alprazolam	5.573 ± 0.010	0.175%	0.175%	15	15
Buprenorphine	9.324 ± 0.0118	2.165%	1.267%	50	50
Mixture Set 2					
Methamphetamine	1.474 ± 0.003	0.154%	0.221%	2.5	5
MDMA	2.039 ± 0.002	0.111%	0.121%	2.5	5
Ketamine	2.638 ± 0.003	0.078%	0.095%	2.5	5
MDMB-INACA	3.700 ± 0.006	0.199%	0.166%	5	5
MDMB-BUTINACA	3.727 ± 0.007	0.208%	0.180%	2.5	5
LSD	6.877 ± 0.011	0.154%	0.166%	50	>50

As for assessing the overall analyte identification accuracy, the library search results of each analyte in the drug class mixture sets were used. The “Match Quality” score of each analyte was obtained from the sample’s extracted mass spectra by using the Wiley Spectral Library and Cayman Spectral Library as reference. The accuracy was examined for concentrations ranging from 1 μg/mL to 50 μg/mL. The Match Quality scores were considered excellent for scores above (90%).

## Results and discussion

### Method development and optimization

The conventional GC-MS methodology has been optimized to a rapid approach, significantly reducing analysis time while maintaining high resolution and sensitivity. The conventional method, characterized by a long temperature ramp from 70°C to 300°C at a rate of 15°C/min with a total run time of 30.33 min, has been shortened in the rapid method to achieve a faster ramp rate of 70°C/min, end in a brief hold at 300°C. This adjustment has reduced the overall run time to merely 10 min. Furthermore, a fixed split injection ratio of 20:1 is employed in both methods, and the same inlet and transfer line temperatures are maintained, ensuring that changes in analytical performance are attributed directly to the modified temperature program rather than variations in sample introduction or ionization conditions. Additionally, the flow rate in the rapid method has been increased to 2 mL/min, compared to 1 mL/min in the conventional method, further contributing to shortening the run time. This enhancement offers a more efficient analysis with potentially reduced degradation of thermally sensitive analytes, thereby extending the method’s applicability to a broader spectrum of volatile compounds.

**FIGURE 1:**
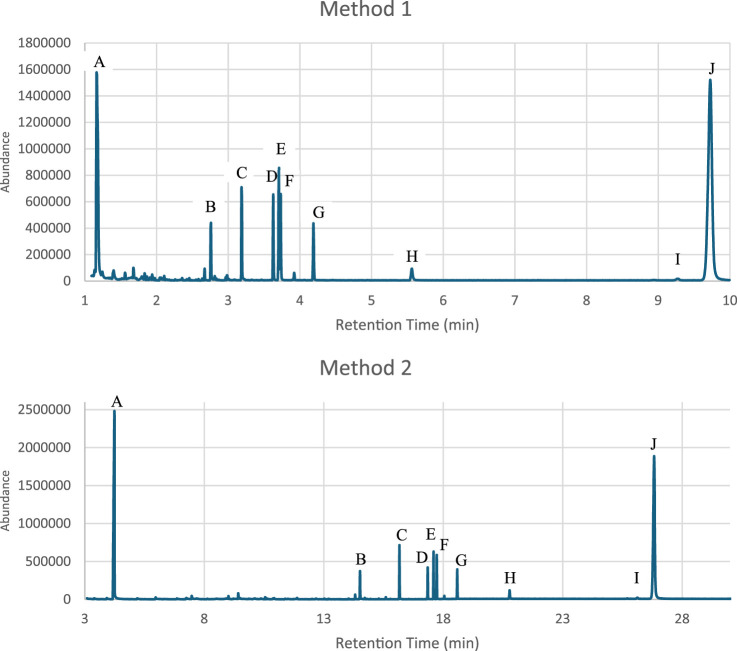
Comparison of the chromatograms from the Mixture Set one analyzed using the rapid GC-MS method “Method 1” and conventional method “Method 2” **(A)** γ-Butyrolactone (GBL) **(B)**. Tramadol **(C)**. Cocaine **(D)**. Codeine **(E)**. Diazepam **(F)**. Δ9-Tetahydrocannabinol (THC) **(G)**. Heroin **(H)**. Alprazolam **(I)**. Buprenorphine **(J)**. Diphenoxylate).

**FIGURE 2:**
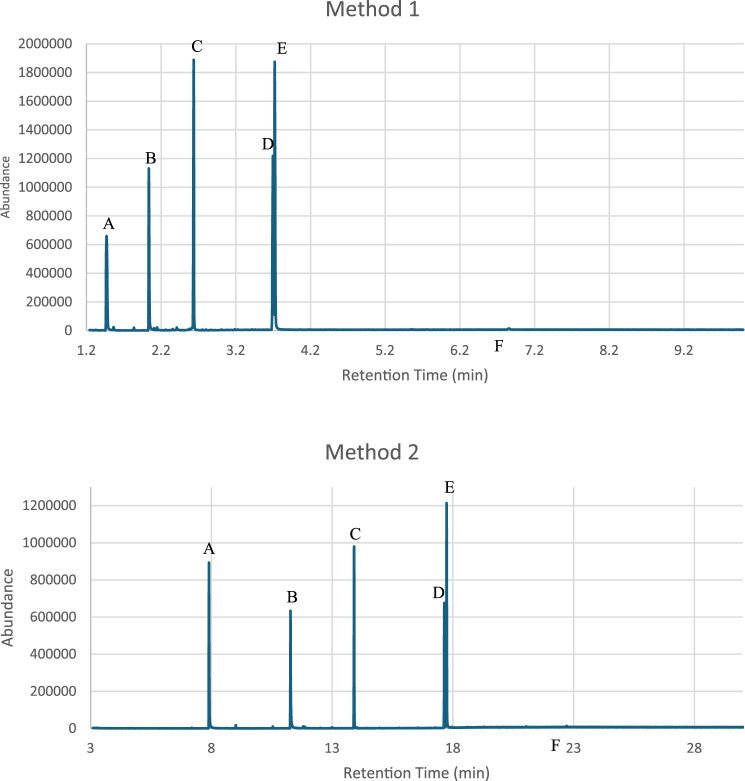
Comparison of the chromatograms from the Mixture Set two analyzed using the rapid GC-MS method “Method 1” and conventional method “Method 2” **(A)**. Methamphetamine **(B)**. MDMA **(C)**. Ketamine **(D)**. MDMB-INACA **(E)**. MDMB-BUTINACA **(F)**. LSD).

The effectiveness of a rapid GC-MS method (Method 1) was evaluated against a conventional GC-MS technique (Method 2) for the analysis of two distinct drug mixture sets, which included various controlled substances. It was clearly demonstrated that the analysis time was significantly reduced by over 60% through Method 1, without compromising the integrity of the results. Enhanced resolution was observed in specific drugs, such as Diazepam and Buprenorphine from Set one and Methamphetamine, MDMA and LSD from Set two when analyzed using the rapid method as shown in Figures 1, 2. Therefore, this advancement is crucial for the precise identification and quantification of substances within complex mixtures, thereby affirming the rapid GC-MS method as a more effective option for urgent forensic cases.

### Method validation

#### Retention time repeatability and reproducibility

The retention time repeatability and reproducibility of the rapid GC–MS method were assessed using test mixtures from Mixture Set 1 (STD1) and Mixture Set 2 (STD2) over 3 weeks, with seven trials conducted per week (n = 21). RTs were determined at the apex of the peaks using MassHunter software, and the average RTs, along with intraday and interday relative standard deviations (RSDs), as summarized in Table 3.

For Mixture Set 1, intraday RSDs ranged from 0.175% to 2.165%, with an average of 0.456%, indicating excellent repeatability. Compounds such as Tramadol, Cocaine, and Diazepam had particularly low RSDs (below 0.25%), while Buprenorphine exhibited the highest intraday RSD of 2.165%, likely due to its longer RT and complex interactions with the GC column. This value slightly exceeds the SWGDRUG recommended threshold of <2% for retention time precision (*SWGDRUG,* 2023) highlighting the need for ongoing instrumental monitoring for this analyte. Interday RSDs ranged from 0.175% to 1.267%, showing similar trends across compounds and reflecting the method’s stability over time.

For Mixture Set 2, intraday RSDs ranged from 0.078% to 0.208%, averaging 0.151%, demonstrating high precision. Ketamine had the lowest intraday RSD at 0.078%, while MDMB-BUTINACA showed a slightly higher value of 0.208%. Interday RSDs for Mixture Set two ranged from 0.095% to 0.221%, with Methamphetamine showing the highest interday RSD, attributed to its shorter RT and sensitivity to instrument fluctuations.

The RSD values for both intraday and interday measurements were within the ±2% threshold recommended by the United Nations Office on Drugs and Crime (UNODC) for low-concentration solutions in seized drug analysis (United Nations Office on Drugs, Crime. Laboratory and Scientific Section, 2009). Figures 1A demonstrates the reproducibility of the RT values obtained for both mixture sets across the seven trials that were conducted over 3 weeks. These results confirm the method’s robustness and reliability for routine forensic applications. However, compounds like Buprenorphine and Methamphetamine warrant careful monitoring due to their slightly higher RSD values.

#### Limits of detection

The LODs for the rapid GC–MS method were determined for analytes in Mixture Set one and Mixture Set two and compared with the conventional GC–MS method. LODs were based on a signal-to-noise ratio of 3:1 and a match score of ≥80% using the Cayman Spectra Library and the Wiley Spectral Library (2021 edition). To contextualize the improvements, the results were benchmarked against previous rapid GC-MS studies such as Capistran and Sisco (2024), who reported LODs ranging from 1 to 10 μg/mL across opioid and benzodiazepine panels, and Bloom et al. (2023), who highlighted challenges in achieving consistent sub-5 µg/mL detection in complex matrices. The current method achieved comparable or improved detection limits across most analytes, reinforcing its suitability for routine screening of seized drug samples in forensic casework.

The rapid GC–MS method exhibited LODs between 1 μg/mL and 50 μg/mL, which were comparable to or better than those of the conventional method. For example, Cocaine had an LOD of 1 μg/mL with the rapid method, significantly outperforming the conventional method’s 2.5 μg/mL. Similarly, Heroin showed an LOD of 5 μg/mL with the rapid method compared to 15 μg/mL with the conventional approach. Notably, LSD was detected at 50 μg/mL using the rapid method but was undetectable with the conventional method. This compound is known to be analytically challenging due to its low volatility, thermolability, and tendency to produce complex fragmentation patterns, often resulting in weaker spectral matches or inconsistent library identification (Tusiewicz, Wachełko, Zawadzki and Szpot, 2024). Other compounds, such as Tramadol, Δ9-tetrahydrocannabinol (THC), Methamphetamine, and MDMA, displayed comparable or slightly improved LODs with the rapid method. Table 3 provides a detailed summary of these results, emphasizing the rapid GC–MS method’s enhanced sensitivity for certain challenging analytes.

#### Analyte identification accuracy

The analyte identification accuracy of the rapid GC–MS method was evaluated for Mixture Set one and Mixture Set two across six concentrations: 1 μg/mL, 2.5 μg/mL, 5 μg/mL, 15 μg/mL, 30 μg/mL, and 50 μg/mL. Match scores, classified as excellent (≥90%), good (80%–89%), fair (60%–79%), or false matches (<60%), were determined using the Cayman Spectra Library and the Wiley Spectral Library (2021 edition). Both libraries cover the analytes assessed in both mixture sets and are generally excellent for drug analysis. Cayman is particularly useful for identifying synthetic drugs and newly emerging psychoactive chemicals, whereas Wiley provides extensive coverage of illicit substances. Due to their complementing functions, using both libraries together yields the most thorough and accurate identification. Notably, using other libraries could influence match scores as differences in spectral resolution, data quality, acquisition conditions, and scoring algorithms among libraries might impact analyte identification accuracy. Certain compounds are absent from some libraries, which results in inaccurate matches.

**FIGURE 3:**
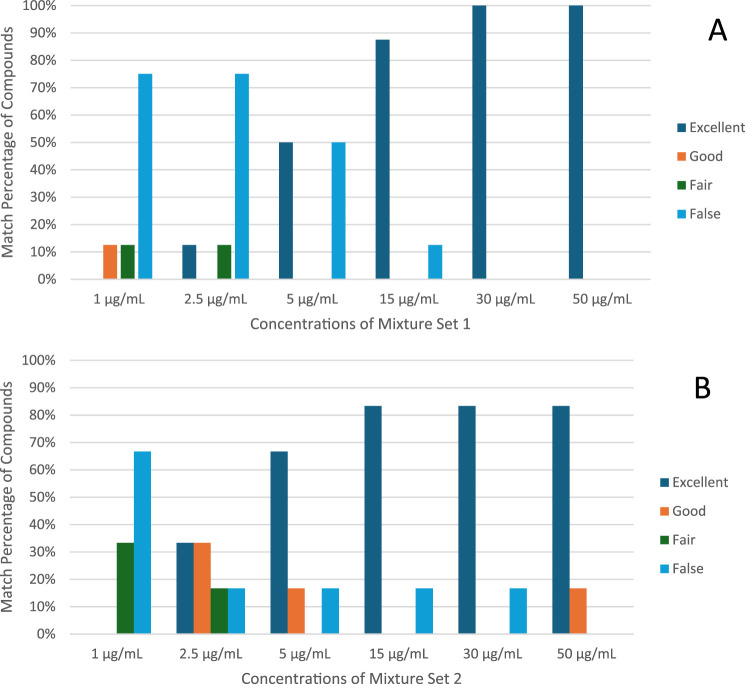
Summary of match scores classification **(A)**. Mixture set 1 (1–50 μg/mL) **(B)**. mixture set 2 (1–50 μg/mL).

Figure 3 illustrates the improvement in match score with increasing concentration. For both mixture sets, only one compound was misidentified at a concentration of 15 μg/mL, and therefore 87% of all analytes in STD1 and 83% of all analytes in STD2 were successfully identified with an exceptional match score. This proves how remarkable the rapid GC-MS method is when it comes to identifying analytes accurately even at lower concentrations.

To elaborate further, for Mixture Set 1, Cocaine consistently achieved excellent identification across all concentrations, with scores reaching 99% at 2.5 μg/mL and above. Similarly, Tramadol, Diazepam, THC, and Codeine attained excellent classification at 5 μg/mL and higher, with match scores exceeding 90%. However, Heroin and Alprazolam showed reduced accuracy at lower concentrations, achieving excellent identification only at 5 μg/mL and 15 μg/mL, respectively. Buprenorphine required higher concentrations, with reliable identification beginning at 30 μg/mL and scores improving to 99% at 50 μg/mL.

For Mixture Set 2, At lower concentrations (1 μg/mL), MDMA, MDMB-INACA, MDMB-BUTINACA, and LSD were classified as false matches due to reduced spectral similarity. Methamphetamine and Ketamine, on the other hand, demonstrated fair results at that concentration. Incrementally, Methamphetamine, Ketamine, MDMA, and MDMB-BUTINACA demonstrated excellent identification at 5 μg/mL and above. LSD posed challenges, achieving good identification only at 50 μg/mL, with a maximum match score of 83% at this concentration.

The rapid GC–MS method consistently delivered high identification accuracy for most analytes at concentrations of 5 μg/mL and above, particularly for compounds like Cocaine, Tramadol, THC, and Ketamine. However, higher concentrations were necessary for reliable identification of challenging analytes such as LSD and Buprenorphine. These findings, detailed in Table 4 underscore the rapid method’s effectiveness for accurate and robust drug identification across diverse substances.

**TABLE 4:** Analyte identification accuracy of mixture set one and mixture set 2.

Mixture set 1								
PPM	Tramadol	Cocaine	Codeine	Diazepam	THC	Heroin	Alprazolam	Buprenorphine
1	40%	86%	0%	0%	68%	0%	0%	0%
2.5	87%	99%	42%	99%	92%	58%	0%	0%
5	93%	99%	99%	99%	99%	97%	93%	0%
15	95%	99%	99%	99%	99%	99%	99%	0%
30	96%	99%	99%	99%	99%	99%	99%	59%
50	95%	99%	99%	99%	99%	99%	99%	99%

Mixture set 2						
PPM	Methamphetamine	MDMA	Ketamine	MDMB-INACA	MDMB-BUTINACA	LSD
1	64%	0%	72%	0%	38%	0%
2.5	83%	83%	90%	72%	96%	0%
5	90%	96%	99%	87%	99%	0%
15	90%	97%	99%	98%	99%	0%
30	90%	97%	99%	99%	99%	50%
50	90%	97%	99%	99%	99%	83%

#### Carryover

**FIGURE 4:**
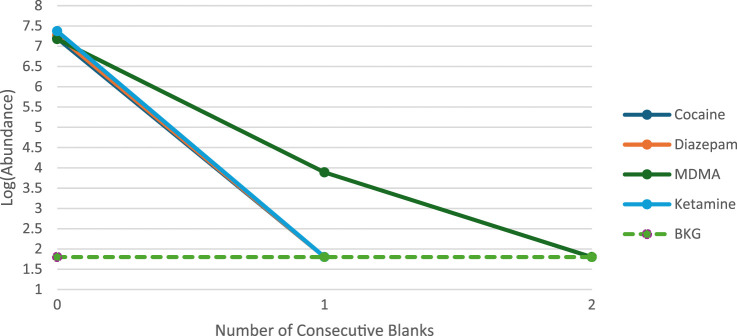
Summary of carryover results at 5 mg/mL.

The presence of analytes in a blank or sample has legal ramifications that can influence the outcome of a criminal prosecution. Hence, instrument carryover was a crucial parameter assessed to validate the rapid GC-MS technique for forensic drug detection. The substances in these mixtures were selected as they are often noticed to create carryover using the conventional GC-MS method since they are usually present in bulk samples at high concentrations. Two concentrated mixtures at 1 mg/mL and 5 mg/mL were examined in order to evaluate this validation criterion. The first mixture consists of two analytes from STD1, Cocaine and Diazepam. Correspondingly, the two analytes in the second mixture are Ketamine and MDMA from STD2. The carryover of analytes was studied by injecting the mixtures at both concentrations followed by five blank samples containing only Methanol. Following the analysis of the solutions at a concentration of 1 mg/mL, no detectable carryover was observed in the subsequent blanks, indicating the absence of analyte residues. Conversely, at a concentration of 5 mg/mL, minimal carryover was detected for one analyte within the second mixture. Notably, Cocaine, Diazepam, and Ketamine exhibited no carryover effects. However, MDMA demonstrated carryover, detectable solely in the first blank post-injection of the mixture. The relative carryover was quantified by comparing the peak area of MDMA from the injection mixture to its peak area in the subsequent blank, yielding a carryover ratio of 0.07%. The peak height of all four analytes in the mixtures were displayed on a log scale in Figure 4. The peak height of the MDMA carryover, as shown in the consecutive blank, exceeded a 3:1 signal-to-noise ratio, described by a dashed line representing average background noise. Subsequent to this initial blank, no traces of MDMA were detected in the following blanks. This observation suggests that the instrument contributes minimally to potential carryover, which can be avoided by maintaining sample concentrations below 5 mg/mL. Importantly, the observed carryover ratio of 0.07% falls well below internationally accepted thresholds. For instance, the UNODC recommends that carryover should not exceed 1% of the analyte signal in blanks, and SWGDRUG guidelines state that no significant analyte peaks should be present in blanks following high-concentration injections (SWGDRUG, 2023; UNODC, 2021), supporting the acceptability of the result within forensic standards. This study’s use of four chemicals to evaluate carryover is considered a limitation, in which a more thorough understanding might be obtained by using larger number of analytes. However, the complexity of mixture preparation at high-concentration and issues with solubility make it technically difficult to utilize multiple analytes. Consequently, the evaluation was restricted to a more manageable and smaller set of chemicals where future work could focus on expanding this analysis to include more analytes to better define carryover behavior across various chemical classes.

### Blind sampling (cases)

In this study, both conventional and rapid GC-MS methods were evaluated to analyze 20 drug samples seized in various criminal cases. These samples included 10 bulk samples containing solid forms of drugs and 10 trace samples from swabs of digital scales, syringes, and other drug-related items. The analysis aimed to compare the identification capabilities of these two methods under real-life criminal cases and assess the effectiveness of the rapid GC-MS approach.

To validate the rapid GC-MS method, mixtures of drug samples that had been previously identified using conventional GC-MS were analyzed. This comparison aimed to verify the consistency and reliability of drug identification between the two methods. To ensure instrument suitability and prevent cross-contamination, blank methanol samples were injected before each analyzed case sample. Additionally, routine system suitability checks were conducted by regularly monitoring instrument performance through blank injections, ensuring no significant carryover was observed. Standard instrument cleaning procedures, including inlet liner replacement and regular column conditioning, were performed periodically according to laboratory protocols to maintain analytical reliability. Following these injections, library searches were conducted to accurately identify the compounds present in the case samples.

Identical compound identifications were yielded by all samples across both methods. This included a range of substances such as THC, Tramadol, Cocaine, Alprazolam, Heroin, Ketamine, LSD, MDMB-INACA, and Buprenorphine in the bulk samples, and multiple cases of Cocaine and Methamphetamine in the trace samples as shown in Table 5. Notably, the rapid GC-MS method was found to significantly reduce the analysis time; The average time taken per case was 10 min, compared to 30 min with the conventional method. This represents a significant reduction in processing time by approximately 66%, enhancing the efficiency of forensic drug analysis. Furthermore, the retention times (RT) and retention time deviations (% RTD) from standard RT observed in the rapid GC-MS method were minimal, indicating its precision. For instance, Cocaine identified in case 256283 using rapid GC-MS had an RT of 3.265 min with a deviation of only 2.383% from the standard, highlighting the method’s accuracy. The MS library match scores, primarily at 99%, further validated the accuracy of the rapid GC-MS method. Such high match scores are crucial in such cases for ensuring the reliability of evidence used in legal contexts. Moreover, the detections of methamphetamine in trace samples showed the method’s sensitivity, which has been further clarified by including the corresponding peak area data in the appendix. Despite the complex matrices associated with trace samples, consistent RTs and acceptable deviations were provided by rapid GC-MS, highlighting its applicability in forensic scenarios where sample quality may vary.

**TABLE 5:** Comparison of Drug Identification by Conventional and Rapid GC-MS in real life criminal cases.

Case number	Case type	Compound identified by conventional GC-MS	Compounds identified by rapid GC-MS	Compound RT (min)	% RTD from standard RT	MS library match score
251116	Bulk	THC (hashish)	THC (hashish)	3.726	0.188	99
251116	Bulk	THC (marijuana)	THC (marijuana)	3.732	0.027	99
257848	Bulk	Tramadol	Tramadol	2.764	0.218	97
256283	Bulk	Cocaine	Cocaine	3.265	2.383	99
258452	Bulk	Alprazolam	Alprazolam	5.568	0.090	99
257764	Bulk	Heroin	Heroin	4.198	0.119	99
256283	Bulk	Ketamine	Ketamine	2.642	0.152	99
255332	Bulk	LSD	LSD	6.873	0.058	99
258042	Bulk	MDMB-INACA	MDMB-INACA	3.726	0.703	99
255722	Bulk	Buprenorphine	Buprenorphine	9.285	0.418	99
251116	Trace	Cocaine	Cocaine	3.19	0.031	99
256283	Trace	Cocaine	Cocaine	3.19	0.031	99
257764	Trace	Heroin	Heroin	4.192	0.024	99
255332	Trace	Methamphetamine	Methamphetamine	1.499	1.696	90
255655	Trace	Methamphetamine	Methamphetamine	1.488	0.950	90
255655	Trace	Methamphetamine	Methamphetamine	1.499	1.696	90
258180	Trace	Methamphetamine	Methamphetamine	1.505	2.103	90
258551	Trace	Methamphetamine	Methamphetamine	1.511	2.510	90
255729	Trace	Methamphetamine	Methamphetamine	1.493	1.289	90
258954	Trace	Methamphetamine	Methamphetamine	1.489	1.018	90

## Conclusion

In conclusion, the developed rapid GC-MS method presented in this study significantly enhances the efficiency of forensic drug analysis by reducing the total analysis time from 30 to 10 min without compromising the analytical integrity essential for legal contexts. This methodological advancement not only meets the increasing demands for quick forensic responses but also maintains high sensitivity and specificity necessary for accurate drug identification. The validation of this rapid approach through systematic studies on repeatability, reproducibility, and limits of detection emphasize its reliability and applicability in real-world forensic settings. Moreover, the method’s ability to provide accurate results with shorter analysis times represents a crucial development in addressing the backlog of cases in forensic laboratories, thereby supporting the broader objectives of law enforcement and public safety. Future work should focus on expanding the application of this method to a broader range of substances and further optimizing the operational parameters to enhance throughput and minimize potential carryover effects. This study provides a robust foundation for the forensic community to adopt rapid GC-MS technologies with confidence, ultimately contributing to more efficient and effective judicial processes.

## Data Availability

The original contributions presented in the study are included in the article/Supplementary Material, further inquiries can be directed to the corresponding author.
