# Supplementary material for: Rapid GC-MS method for screening seized drugs in forensic investigations: optimization and validation
Source: Front Chem. 2025 Jun 10;13:1559279. doi: 10.3389/fchem.2025.1559279 (PMC12185466; doi:10.3389/fchem.2025.1559279)
Supplement: Supplementary file 1 [file DataSheet1.docx]

Appendix

*Figure 1A. Retention Time Precent Data for rapid GC-M method A. Mixture set 1 B. mixture set 2.*

*Figure 2A. Calibration Curve A. Mixture set 1 (1-50 µg/mL) B. mixture set 2 (1-50 µg/mL).*

*Figure 3A. Total ion chromatogram of case number 255655 analyzed using the rapid GC-MS method (Compound Identified: Methamphetamine).*

*Figure 4A. Total ion chromatogram of case number 257484 analyzed using the rapid GC-MS method (Compound Identified: Tramadol).*

*Figure 5A. Total ion chromatogram of case number 257764 analyzed using the rapid GC-MS method (Compound Identified: Heroin).*

*Figure 6A. Total ion chromatogram of case number 256283 analyzed using the rapid GC-MS method (Compound Identified: Cocaine).*

*Figure 7A. Total ion chromatogram of case number 251116 analyzed using the rapid GC-MS method (Compounds Identified: Cannabidiol “CBD”, Delta-9-tetrahydrocannabinol “THC”, Cannabinol “CBN”).*

*Figure 8A. Retention Time percentage deviation across all analytes in the cases.*

*Figure 9A. Peak area of all the trace cases.*
